# Homology Modeling and Virtual Screening to Discover Potent Inhibitors Targeting the Imidazole Glycerophosphate Dehydratase Protein in *Staphylococcus xylosus*

**DOI:** 10.3389/fchem.2017.00098

**Published:** 2017-11-10

**Authors:** Xing-Ru Chen, Xiao-Ting Wang, Mei-Qi Hao, Yong-Hui Zhou, Wen-Qiang Cui, Xiao-Xu Xing, Chang-Geng Xu, Jing-Wen Bai, Yan-Hua Li

**Affiliations:** ^1^College of Veterinary Medicine, Northeast Agricultural University, Harbin, China; ^2^Heilongjiang Key Laboratory for Animal Disease Control and Pharmaceutical Development, Harbin, China; ^3^College of Science, Northeast Agricultural University, Harbin, China

**Keywords:** imidazole glycerophosphate dehydratase (IGPD), *Staphylococcus xylosus*, homology model, virtual screening, biofilm formation

## Abstract

The imidazole glycerophosphate dehydratase (IGPD) protein is a therapeutic target for herbicide discovery. It is also regarded as a possible target in *Staphylococcus xylosus* (*S. xylosus*) for solving mastitis in the dairy cow. The 3D structure of IGPD protein is essential for discovering novel inhibitors during high-throughput virtual screening. However, to date, the 3D structure of IGPD protein of *S. xylosus* has not been solved. In this study, a series of computational techniques including homology modeling, Ramachandran Plots, and Verify 3D were performed in order to construct an appropriate 3D model of IGPD protein of *S. xylosus*. Nine hits were identified from 2,500 compounds by docking studies. Then, these nine compounds were first tested *in vitro* in *S. xylosus* biofilm formation using crystal violet staining. One of the potential compounds, baicalin was shown to significantly inhibit *S. xylosus* biofilm formation. Finally, the baicalin was further evaluated, which showed better inhibition of biofilm formation capability in *S. xylosus* by scanning electron microscopy. Hence, we have predicted the structure of IGPD protein of *S. xylosus* using computational techniques. We further discovered the IGPD protein was targeted by baicalin compound which inhibited the biofilm formation in *S. xylosus*. Our findings here would provide implications for the further development of novel IGPD inhibitors for the treatment of dairy mastitis.

## Introduction

Bovine mastitis, a multi-factorial disease, causes enormous economic losses in the dairy industry worldwide (Anderson and Azizoglu, 2014). Studies have shown that some bacteria strains, such as *Staphylococcus aureus* (*S. aureus*) (Vasudevan et al., 2003), *Streptococcus agalactiae* (Boonyayatra and Pata, 2016), *Escherichia coli* (Silva et al., 2014), are the common pathogens that cause mastitis infections in dairy cows. Besides, Tenhagen et al. (2006) (Vasudevan et al., 2003) have reported that *Staphylococcus xylosus* (*S. xylosus*) isolated from a bovine mastitis could also cause the mastitis infection in dairy cows. *S. xylosus* is also *one* of the coagulase-negative staphylococci (CoNS) (Osman et al., 2016), which has strong ability of biofilm formation (Planchon et al., 2006, 2009). In addition, the L-histidine synthesis pathway is involved in the formation of biofilm in *S. xylosus* (Xu et al., 2017). The imidazole glycerophosphate dehydratase (IGPD), one of the specific enzymes, catalyzes the dehydration of imidazoleglycerol phosphate (IGP) to imidazoleacetol phosphate (IAP) (Hawkes et al., 1995), which synthesize L-histidine in the synthesis pathway (Dietl et al., 2016).

Currently, the IGPD protein becomes an attractive target for herbicide discoveries due to a necessary role in the histidine biosynthesis (Ahangar et al., 2013). Screening compounds via high-throughput virtual techniques require the 3D structure of a protein. However, the 3D structure of IGPD protein of *S. xylosus* has not been solved yet. Generally, it has been implemented that a protein sequence with 30% identity to a known structure is considered to be a threshold limit for the accuracy of homology modeling (Xiang, 2006; Henriksen et al., 2010). Recently, the 3D crystal structures of IGPD protein from *S. aureus* (Henriksen et al., 2010), *Arabidopsis thaliana* (*A. thaliana*) (Bisson et al., 2015), *Cryptococcus neoformans* (*C. neoformans*) (Chaudhuri et al., 2001) have been solved and are available in the protein databank, which provide us an opportunity to construct the 3D structure of IGPD of *S. xylosus*.

Computational methods e.g., virtual screening is an attractive and cost-effective way (Sakkiah et al., 2011) to identify potential novel inhibitors against a target protein (Zhu et al., 2007). If the structure of an active compound is available, the computational techniques could help to reduce the number of candidates for experimental validations. Docking-based virtual screening approach is one of the widely used methods to screen active compounds against the IGPD protein.

In this study, after constructing the 3D structure of the IGPD protein, we screened some of the best active compounds and verified their activities against the formation of biofilm in *S. xylosus*. Nine potential hits were identified using the docking-based virtual screening. The compounds inhibiting biofilm formation of *S. xylosus* were evaluated by crystal violet staining. One of the compounds, baicalin has shown to significantly inhibit the biofilm formation in *S. xylosus in vitro*.

## Materials and methods

### Homology modeling of the IGPD protein

To date, the 3D structure of the IGPD protein from *S. xylosus* is not available. In order to construct the 3D structure of the IGPD protein, the protein of homology modeling technique was used for docking and structure-based design. It is pivotal to construct the 3D IGPD structure based on the known structures by homology modeling method. The amino acid sequences of the IGPD protein were isobarically tagged for relative and absolute quantitation (iTRAQ) labeling (Xu et al., 2017; Table 1). Hence, sequence similarity searches were carried out by utilizing BLASTp analysis (Altschul et al., 1990), which revealed suitable templates for the homology modeling. Target sequences of the IGPD protein were constructed based on the build homology models using Discovery Studio (version 3.0), (PS)2-v2: protein structure prediction server (Chen et al., 2006, 2009), as well as MODELLER (Webb and Sali, 2016). The needle pairwise sequence alignment was used to calculate sequence similarity (Li et al., 2015). Sequences identity and similarity from multiple sequence alignments were further confirmed by ClustalW (Larkin et al., 2007). Secondary structure matching (SSM) (Krissinel and Henrick, 2004) was used for superposition of protein structures (Bauer et al., 2008). Finally, the best model with the lowest probability density functions (PDF) total energy and Discrete Optimized Potential Energy (DOPE) score were selected (Shen and Sali, 2006).

**Table 1:** The full-length protein sequence of IGPD from the gene sequence.

MIYQKTRNTAETQLSISLADDNRPSKINTGVGFLDHMLTLFTFHSNLSITIEANGDTEV DDHHVTEDIGIVLGQLLLEMTRERKSFQRYGVSYIPMDETLARTVVDISGRPFLSFNA HLSREKVGTFDTELVEEFFRALVINARLTTHIDLIRGGNTHHEIEGIFKSFARALKESLS SNDIDGTPSSKGVIE

### Homology model validation

The model was evaluated using Ramachandran Plot (Kar et al., 2013), and was verified through the Profile-3D analysis. The qualified models were evaluated using the Qualitative Model Energy Analysis (QMEAN) (Benkert et al., 2008, 2009).

### Molecular docking of compounds

After the initial model evaluations, molecular docking was carried out using the Discovery Studio (version 3.0). A set of 2,500 compounds from the PubChem Compound Database (https://pubchem.ncbi.nlm.nih.gov/), such as flavonoids, anthraquinones, and benzanthrone were used for the preparation of ligands to generate 3D conformations. The ADMET prediction was used to filter the water-insoluble drugs. The new 3D IGPD protein structure was then used in the docking study. All hydrogen atoms were added to the protein using DS 3.0. The Charmm Force Field was assigned, and the active site was defined. Then, the optimized ligands docked with the homology protein using the DS-CDOCKER protocol. The Pose Cluster Radius was set up to 0.5 and other parameters were used the default settings. Finally, the first hits by virtual screening were further evaluated by biofilm formation assays *in vitro*.

### Biofilm formation

#### Bacterial strains and culture conditions

The *S. xylosus* ATCC700404 strain was used in this study (Yang et al., 2016). *S. xylosus* was cultured in Trypticase Soy Broth (TSB) (TSB: Summus Ltd, Harbin, Heilongjiang, China) at 37°C for 12 h. Then, all bacterial strains were repeatedly subcultured under the same conditions. The final cultures were used for the minimal inhibitory concentration (MICs) assays and the biofilm formation assays.

#### Inhibitory activity of compounds against the IGPD

The identified compounds used for MICs assays were determined three times using the protocol described previously (Yang et al., 2016). Briefly, the overnight cultures of *S. xylosus* were diluted to a density of McFarland 0.5 standard [corresponding to 1 × 10^8^ colony-forming units (CFU)/ml]. Then, the cultures of *S. xylosus* were diluted 1:100 using sterile TSB. Then 100 μl samples were added to each well of a 96-well plate (Corning Costar® 3599 Corning, NY, USA) containing serial dilutions of compounds in 100 μl culture medium. Control bacteria were cultivated in the absence of compounds. The MICs were determined as the lowest concentration of potential compounds after incubation for 24 h at 37°C.

#### Biofilm formation assay

The biofilm formation assay was carried out to evaluate the ability of *S. xylosus* biofilm disruption by the screened compounds using 96-well microtiter plates (Corning Costar® 3599 Corning, NY, USA) (Yang et al., 2016). Negative control wells contained broth only. Positive control wells contained culture medium and bacterial suspension. Biofilms were treated as described above (Yang et al., 2016). Briefly, the plates were covered and incubated aerobically for 24 h at 37°C. Then, the content of each well was aspirated and washed three times with 0.25 ml of sterile physiological saline. The remaining attached bacteria were fixed with 0.2 mL of 99% methanol (Guoyao Ltd, China) per well. After 15 min fixation plates were emptied and dried at 37°C. Then, the plates were stained for 5 min with 0.2 mL of 2% crystal violet (Guoyao Ltd., China). The excess stain was rinsed off by placing the plate under running tap water. After the plates were air dried, the dye was resolubilized with 0.16 mL of 33% (*v/v*) glacial acetic acid (Guoyao Ltd, China) per well. The OD of each well was measured at 570 nm using a microtiter plate reader (DG5033A, Huadong Ltd., Nanjing, Jiangsu, China).

#### Scanning electron microscopy

The biofilm of *S. xylosus* was examined by an electron microscopy (FEI Quanta, Netherland), which was described previously (Yang et al., 2016). Briefly, the overnight cultures of *S. xylosus* were diluted in the sterile THB supplemented with 5 % (*v/v*) fetal bovine serum (corresponding to 1 × 10^8^ colony-forming units/ mL). Then, the overnight bacterial cultures were diluted with 5 mg/mL baicalin or without baicalin and then were added to each well of a six-well plate containing rough glass slide or normal glass slide. The supernatant was removed after 24 h incubation. The biofilms obtained from bacterial cells were prepared for analysis using a scanning electron microscopy.

#### Statistical analysis

All the experiments were performed in triplicates. Data analysis and calculation of standard deviation were performed with SPSS 11.0.0 (IBM, USA).

## Results and discussions

### Homology modeling of the IGPD

**Figure 1:**
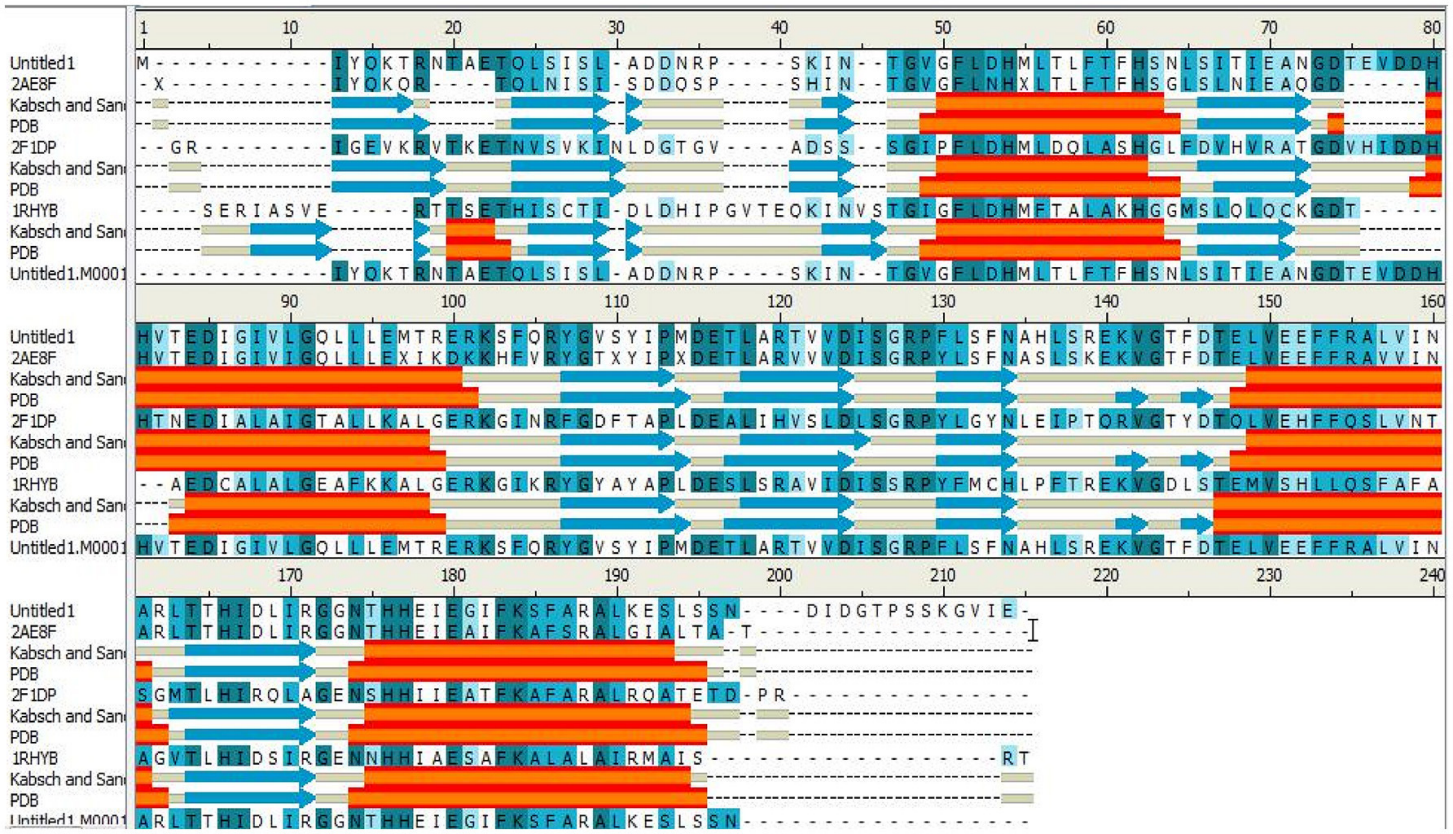
The alignment sequence between IGPD proteins from *Staphylococcus xylosus. Staphylococcus aureus* (PDB ID: 2AE8F), *Arabidopsis thaliana* (PDB ID: 2F1DP) and *Cryptococcus neoformans* (PDB ID: 1RHYB). The α helices are shown as red blocks. The β sheets are shown as blue blocks. Most of the secondary structures are observed to be well conserved.

The IGPD protein is a potential therapeutic target for herbicide discovery. Here, we used a molecular docking technology to discover the IGPD inhibitors. Up to date, the crystal structure of IGPD protein in *S. xylosus* has not been obtained. Thus, the homology modeling was used to build the 3D structure of that protein in this study. Generally, a protein sequence with 30% identity to a known structure is considered to be threshold limit for the accuracy of homology modeling (Xiang, 2006; Henriksen et al., 2010). From the Table 2, the 3D structure of IGPD from *S. aureus* (PDB code: 2AE8F) (Henriksen et al., 2010) was selected as the template (Supplementary Figure 1), which showed 22.8% sequence identity, 46.6% sequence similarity, 74% query cover, and high resolution with 2.01 Å. Further, multiple sequences alignment of IGPD with *S. aureus* (PDB code: 2AE8F), *A. thaliana* (PDB code: 2F1DP) (Henriksen et al., 2010) and *C. neoformans* (PDB code: 1RHYB) (Chaudhuri et al., 2001) were performed using ClustalW (Larkin et al., 2007). The highly conserved region of the structural elements was found, which was shown in Figure 1. Finally, based on the least PDF total energy (4591.1885) and DOPE score (Shen and Sali, 2006) (-19163.410156) the best model (Supplementary Figure 2) was selected and its structural compatibility was verified using several validation servers.

**Table 2:** 3D structures used as templates for homology modeling of IGPD protein.

**Accession**	**Identity**	***e*-value**	**Resolution**
2AE8_F	74	4.04E-71	2.01
2F1D_P	40	4.18E-36	3
1RHY_B	40	6.90E-34	2.3
1ZFJ_A	32	2.783	1.9
2WU8_A	24	3.69587	2.25
2ZUE_A	25	3.98409	2
3K94_B	34	5.60917	2.101

### Homology model validation

**Figure 2:**
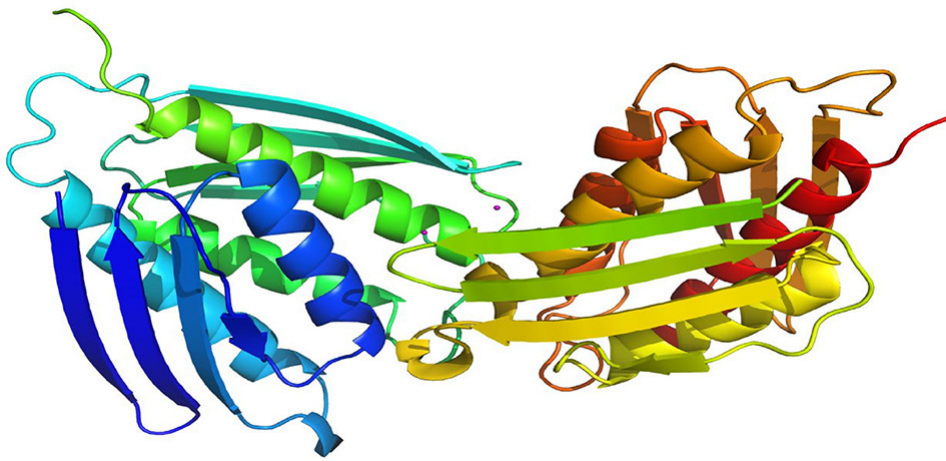
Superposition of ligand-free dimers of IGPD showing the difference flap conformations.

The selected model was then evaluated by Profiles-3D. The calculated verify score for each of residues in the IGPD model was shown in Supplementary Figure 3. The Ramachandran Plot analyzed by RAMPAGE was often used to check the correctness in proteins for the stereochemical feasibility (Gopalakrishnan et al., 2008). The final model was selected (Supplementary Table 1), which had the maximum number of residues in the favored regions (93.26%), minimum disallowed regions (1.69%), and with no significant influence on our modeling and docking studies, since they are far from the active site of the IGPD in Supplementary Figure 4. At the meantime, it was shown to have the best secondary structures in the superposition for IGPD protein (Figure 2). It is also the Superimpose protein that deals with structural superpositions of molecules in a widespread sense (Bauer et al., 2008). Indeed, the *Z*-score calculated for the template was −3.75, which indicated that the overall geometrical quality of the model was within the acceptable range in comparison with the solved experimental structure using the QMEAN. Overall, the validation results demonstrated that the established homology model of the IGPD should be a reasonable structure, and could be used in the following studies.

### Molecular docking of compounds

**Figure 3:**
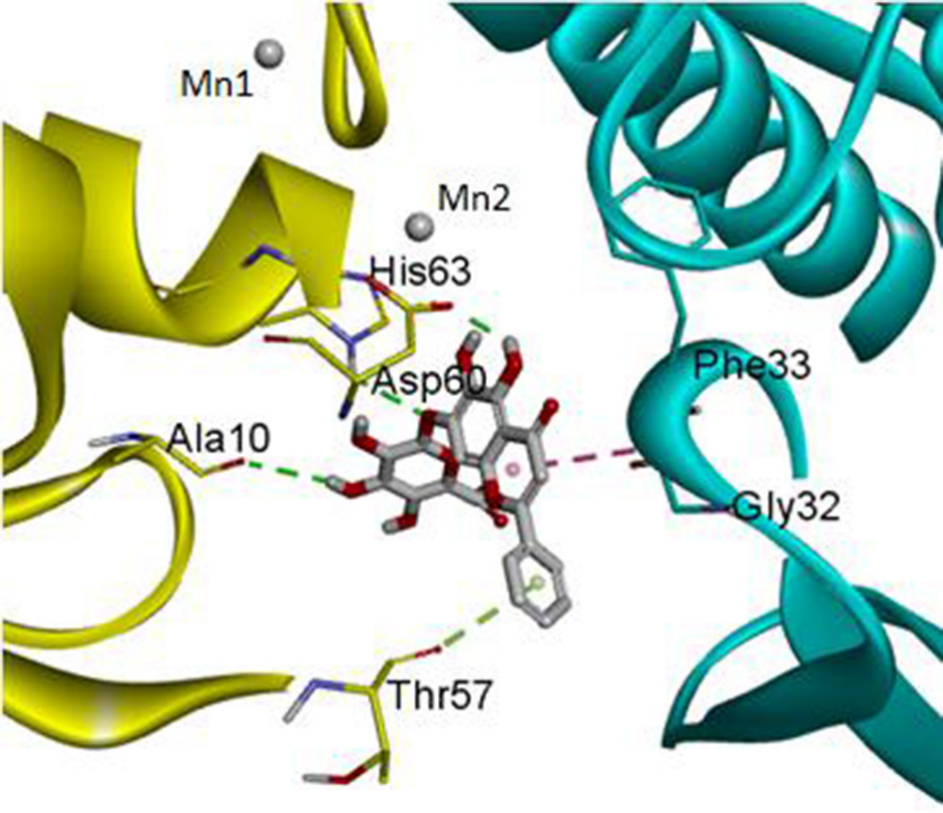
The protein backbone is shown and colored gold, cyan for chains A, A' respectively. The two manganese ions are shown as labeled silvery white spheres, and the atoms of the compound, baicalin, are shown in ball-and-stick format and colored by atom. Polar interactions are shown as dashed colored lines. Among them, the green dotted line represents the hydrogen bond interaction. The pink dotted line represents hydrophobic interaction.

The DS 3.0 was used for the virtual screening of IGPD protein. First of all, 2500 compounds from the PubChem Compound Database were used for the screening, e.g., flavonoids, anthraquinones, and benzanthrone can inhibit the formation of biofilms, especially among the known experiments emodin inhibits *Streptococcus suis* (*S. suis*) biofilm formation (Yang et al., 2015), rutin inhibits *S. suis* biofilm formation (Wang et al., 2017), and caffeic acid derivatives against *Candida albicans* biofilm (De Vita et al., 2014). In order to discover inhibitors targeting the IGPD faster and more accurate, the ADMET prediction was used to eliminate compounds with unfavorable aqueous solubility compounds. The rest of the compounds were employed in the docking study. This contributes to the better interaction of proteins and compounds (Hiremath, 2009). It is conceived that the 3D structure of *A. thaliana* of active site is positioned between the two active-site manganese ions (Mn1 and Mn2) (PDB code: 2F1DP) which has shown to play a key role in the process of the IGPD activity in the previous study (Henriksen et al., 2010; Bisson et al., 2015). In this study, the two active-site manganese ions were coped (Figure 3). Then molecular docking studies were performed to find the optimal conformation in the binding site of the IGPD receptor and to identify the interaction between them, as well as to complement the CDOCKER docking studies for the rational design of compounds. According to the calculated score, nine compounds had the best -CDOCKER ENERGY and -CDOCKER INTERACTION ENERGY scores (Table 3). Among them, baicalin was one of the hits. Currently, it has been shown that the interaction mode analysis of nitrogen-containing heterocyclic compounds was found to be some hydrophobic interactions in the IGPD protein of *Cryptococcus neoformans* (PDB code: 1RHYB) (Shen et al., 2011). However, whether baicalin can inhibit the IGPD protein of *S. xylosus* has not been reported. By docking analysis, baicalin was one of the strongest in attracting the IGPD protein in *S. xylosus* when complexes through specific residual interaction with three H-bonds of His 63, Asp 60, and Ala 10 (Figure 3). In addition, there is one hydrophobic interaction with Gly 32. It also plays an important role in the interaction between the IGPD protein and baicalin. The estimated CDOCKER energy of baicalin was found to be −38.9626 kcal/mol. These effects together indicated that baicalin can inhibit IGPD protein of *S. xylosus*.

**Table 3:** The screen of compounds of MICs and the biofilm formation.

**PubChem CID**	**Name**	**Structure**	**-CDOCKER energy**	**-CDOCKER interation energy**	**MIC (mg/mL)**
64982	Baicalin	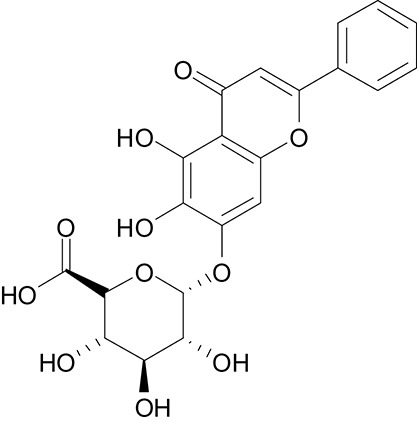	38.9626	58.3956	>5
5281614	Fisetin	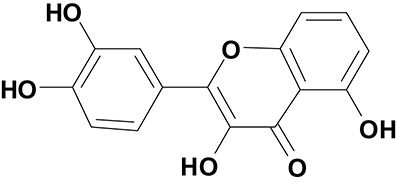	89.0047	56.9835	>12
10639	Physcion	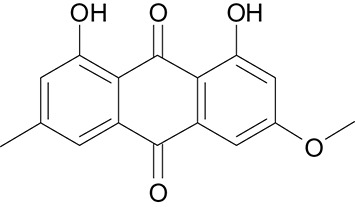	39.4196	38.5684	> 4.5
1794427	Chlorogenic Acid	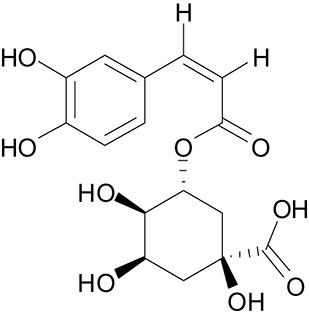	44.9349	66.4344	>9
23711819	Sodium Danshensu	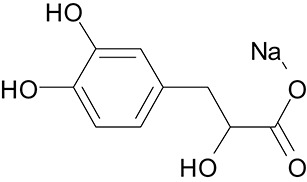	62.4521	58.0722	>21
3220	Emodin	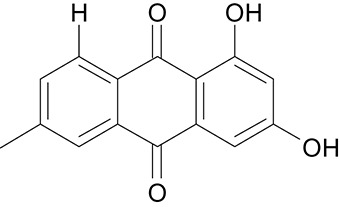	46.6379	39.5454	0.156
10208	Chrysophanol	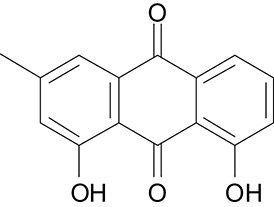	41.7041	38.5834	0.156
445858	Ferulic Acid	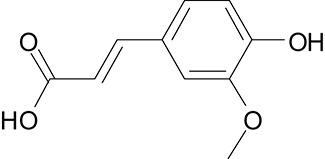	48.0386	51.0912	0.875
5281605	Baicalein	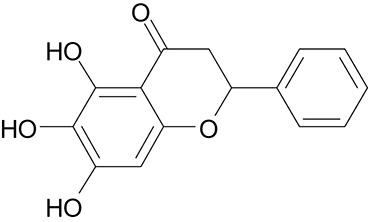	54.3528	45.3041	2

### *In vitro* biofilm formation

**Figure 4:**
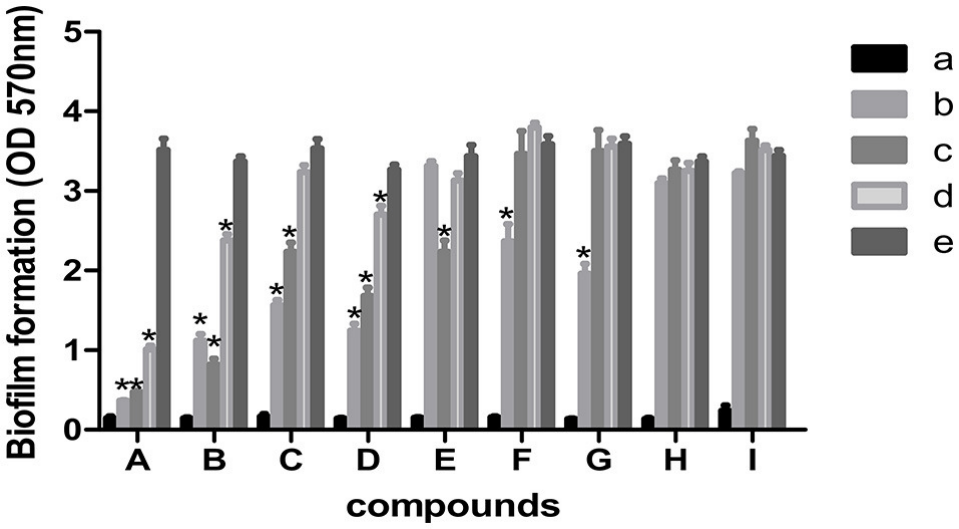
Effect of MICs of compounds at different concentrations on biofilm formation by *Staphylococcus xylosus* ATCC700494. Data are expressed as means ± standard deviations. Asterisk indicates signifcantly different (^*^p < 0.05) compared to untreated control bacteria. **(A)** Baicalin: (a) blank, (b) 5 mg/mL (c) 2.5 mg/mL, (d) 1.25 mg/mL, (e) control. **(B)** Fisetin: (a) blank, (b) 12 mg/mL (c) 6 mg/mL, (d) 3 mg/mL, (e) control. **(C)** Emodin: (a) blank, (b) 1/2 MIC (c) 1/4 MIC, (d) 1/8 MIC, (e) control. **(D)** Ferulic acid: (a) blank, (b) 1/2 MIC (c) 1/4 MIC, (d) 1/8 MIC, (e) control. **(E)** Physcion: (a) blank, (b) 4.5 mg/mL (c) 2.25 mg/mL, (d) 1.125 mg/mL, (e) control. **(F)** Chlorogenic: (a) blank, (b) 9 mg/mL (c) 4.5 mg/mL, (d) 2.25 mg/mL, (e) control. **(G)** Physcion: (a) blank, (b) 1/2 MIC (c) 1/4 MIC, (d) 1/8 MIC, (e) control. **(H)** Baicalein (a) blank, (b) 1/2 MIC (c) 1/4 MIC, (d) 1/8 MIC, (e) control. **(I)** Solidum danshensu: (a) blank, (b) 21 mg/mL (c) 10.5 mg/mL, (d) 5.25 mg/mL, (e) control.

**Figure 5:**
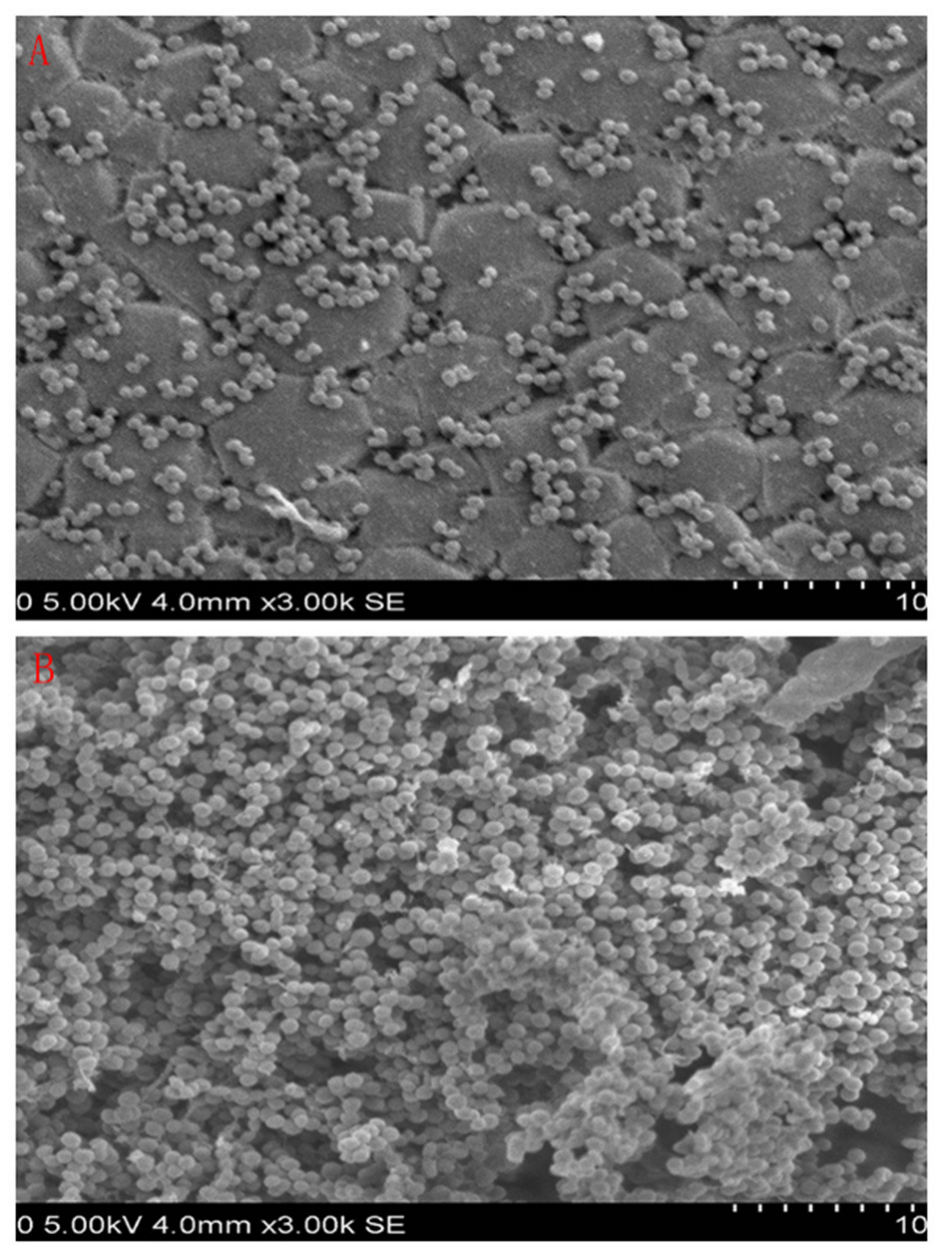
Scanning electron micrographs of *S. xylosus* ATCC700794 biofilm following growth in TSB supplemented with 5 mg/mL of baicalin (a) or control (b). Controls refer to the absence of baicalin. **(A)** 5 mg/mL of baicalin. **(B)** control.

The 9 compounds were evaluated against the *S. xylosus* biofilm formation with different MICs listed in Table 3. The results (Figure 4) showed that baicalin and fisetin had the highest ability to inhibit the formation of *S. xylosus* biofilm. Then, the ability of inhibition decreases progressively from the emodin and ferulic acid to phycsion, chlorogenic acid, and chrysophanol, whereas, the baicalein and sodium danshensu showed the lowest ability to inhibit the formation of *S. xylosus* biofilm. Most recently studies have shown that the inhibitory effect of baicalin on biofilm formation in *Pseudomonas aeruginosa pathogenicity* (Luo et al., 2017) and *Cronobacter sakazakii* (Jing et al., 2016) were 83.7, 66.6%, respectively. Interestingly, we have shown here baicalin can also inhibit *S. xylosus* biofilm formation (73.3%). In addition, we further used the scanning electron microscopy method to confirm this finding. As shown in the control (Figure 5B), a thick biofilm made of aggregates and microcolonies of *S. xylosus* almost completely covered the surface of the rough glass slide. However, when the culture medium was supplemented with 5 mg/mL of baicalin, the individual pairs of *S. xylosus* and the individual short chains of *S. xylosus* were attached to the rough glass slide (Figure 5A). This is the first report that baicalin can inhibit the formation of *S. xylosus* biofilm.

## Conclusions

The IGPD inhibitors in *S. xylosus* are potential new drug targets. We have constructed a 3D structure of IGPD protein. We have discovered 9 compounds that show interaction with IGPD protein. Among them, baicalin showed the highest capacity to inhibit the formation of *S. xylosus* biofilm *in vitro*. The novelty of this work lies in the use of the constructed 3D structure of the IGPD protein to screen compounds inhibiting the biofilm formation of *S. xylosus*, which could accelerate the development of novel compounds for solving the persistent infection of mastitis in dairy cows.

## Author contributions

Y-HL and J-WB designed the whole experiment; X-RC directed the completion of the experiment; X-TW, M-QH, Y-HZ, W-QC, X-XX, and C-GX provided help during the experiment.

### Conflict of interest statement

The authors declare that the research was conducted in the absence of any commercial or financial relationships that could be construed as a potential conflict of interest.
