# Supplementary material for: Homology Modeling and Virtual Screening to Discover Potent Inhibitors Targeting the Imidazole Glycerophosphate Dehydratase Protein in Staphylococcus xylosus
Source: Front Chem. 2017 Nov 10;5:98. doi: 10.3389/fchem.2017.00098 (PMC5686052; doi:10.3389/fchem.2017.00098)
Supplement: Supplementary file 1 [file Table1.docx]

Supplementary Material

**Homology modeling and virtual screening to discover potent inhibitors targeting the imidazole glycerophosphate dehydratase protein in** ***Staphylococcus xylosus***

*Xing-Ru Chen^1,3^, Xiao-Ting Wang^1,3^, Mei-Qi Hao^1,3^, Yong-Hui Zhou^1,3^, Wen-Qiang Cui^1,3^, Xiao-Xu Xing^1,3^,Chang-Geng Xu^1,3^, Jing-Wen Bai****^2^****^*^, Yan-Hua Li^1,3*^*

*^1College of Veterinary Medicine, Northeast Agricultural University, Harbin, China,^*

*^2College of Science, Northeast Agricultural University, Harbin, China,^*

*^3Heilongjiang Key Laboratory for Animal Disease Control and Pharmaceutical Development, Harbin, China^*

Correspondence author:

Professor Jingwen Bai, College of Science, Northeast Agricultural University, 600 Changjiang Road, Xiangfang, Harbin, Heilongjiang 150030, P.R. China

Tel：+86 451 55191810

E mail:baijingwen@neau.edu.cn (J.-W. Bai).

Professor Yanhua Li, College Veterinary Medicine, Northeast Agricultural University, 600 Changjiang Road, Xiangfang, Harbin, Heilongjiang 150030, P.R. China

Tel：+86 451 55191881

E mail: liyanhua1970@163.com (Y.-H.Li ).

**Supplementary Figures**

**
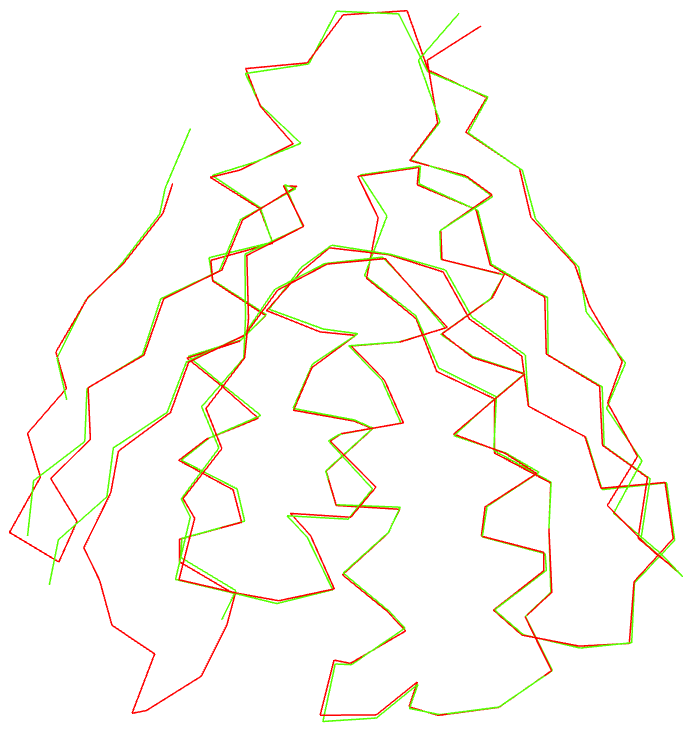
**

**Supplementary Figure 1.** Superposition of template (2AE8F) colored with green and IGPD model colored with red.


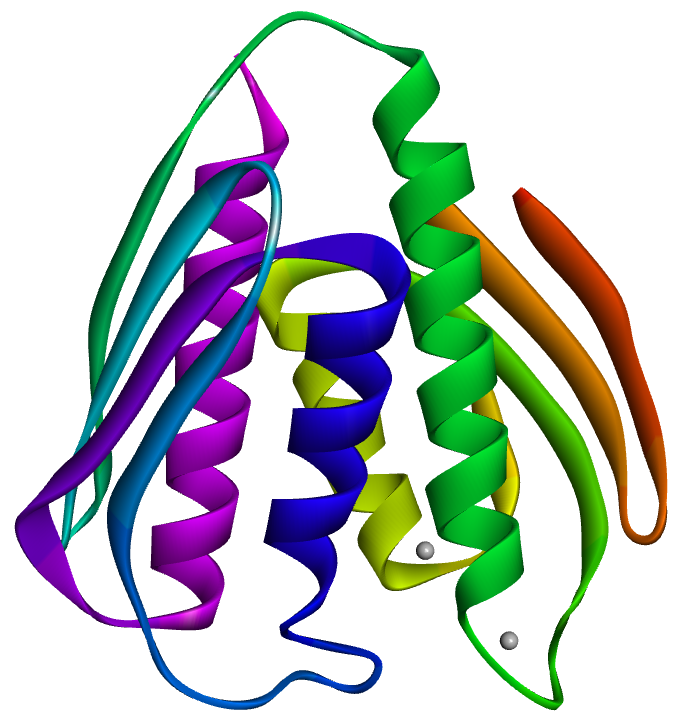


**Supplementary Figure 2.** Modeled structure of IGPD protein from *Staphylococcus xylosus.*

*
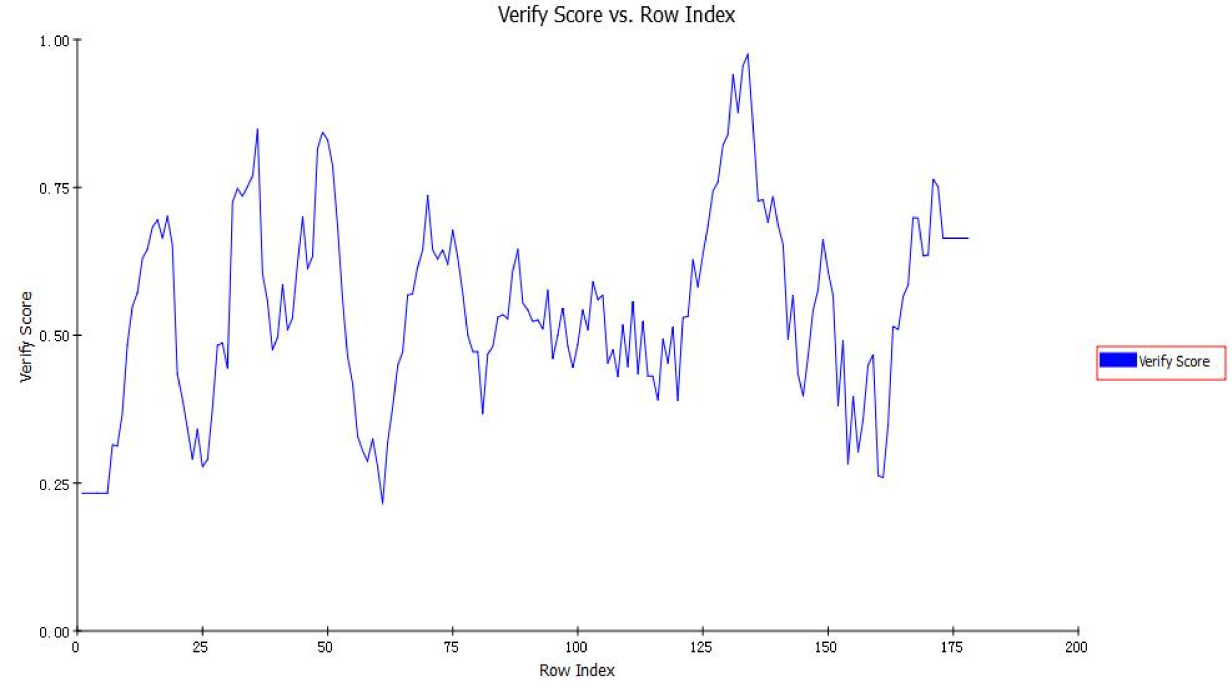
*

**Supplementary Figure 3.** The IGPD amino acid score of each sequence.


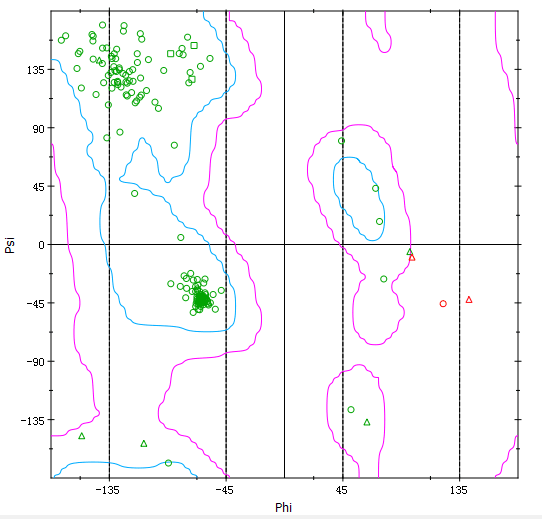


**Supplementary Figure 4.** Ramachandran Plot:The red color is indicative of most favored region , however, yellow and light brown showed additionally allowed and generously allowed regions, respectively.

**Supplementary Tables**

**Supplementary Table 1.** Ramachandran Plot quality of the models generated from Discovery Studio (version 3.0), (PS)2-v2: Protein Structure Prediction Server, and MODELLER.

| Protein | Program | Number of residues | Ramachandran Plot(%) | | | QMEAN |
| --- | --- | --- | --- | --- | --- | --- |
|  |  |  | Favored | Allowed | Disallowed |  |
| IGPD | Discovery Studio (version 3.0) | 178 | 0.9326 | 0.0506 | 0.0169 | -3.75 |
|  | (PS)2-v2 | 192 | 0.9323 | 0.0521 | 0.0156 | -2.31 |
|  | MODELLER | 171 | 0.9415 | 0.0409 | 0.0175 | -1.776 |
